# Is HELICS the Right Way? Lack of Chest Radiography Limits Ventilator-Associated Pneumonia Surveillance in Wales

**DOI:** 10.3389/fmicb.2016.01271

**Published:** 2016-08-18

**Authors:** Richard Pugh, Wendy Harrison, Susan Harris, Hywel Roberts, Gareth Scholey, Tamas Szakmany, the WICSARG Investigators and WHAIP, Ceri Battle

**Affiliations:** ^1^Department of Anaesthetics, Glan Clwyd HospitalBodelwyddan, Wales; ^2^Public Health Wales, Temple of Peace and HealthCardiff, Wales; ^3^Adult Critical Care Services, University Hospital WalesCardiff, Wales; ^4^Cardiff Institute of Infection and Immunity, Cardiff UniversityCardiff, Wales; ^5^Directorate of Critical Care, Royal Gwent HospitalNewport, Wales

**Keywords:** ventilator-associated pneumonia, ventilator-associated tracheobronchitis, respiratory tract infection, ventilator-associated event, ventilator-associated complication, surveillance

## Abstract

**Introduction:** The reported incidence of ventilator-associated pneumonia (VAP) in Wales is low compared with surveillance data from other European regions. It is unclear whether this reflects success of the Welsh healthcare-associated infection prevention measures or limitations in the application of European VAP surveillance methods. Our primary aim was to investigate episodes of ventilator-associated respiratory tract infection (VARTI), to identify episodes that met established criteria for VAP, and to explore reasons why others did not, according to the Hospitals in Europe Link for Infection Control through Surveillance (HELICS) definitions.

**Materials and Methods:** During two 14-day study periods 2012–2014, investigators reviewed all invasively ventilated patients in all 14 Welsh Intensive Care Units (ICUs). Episodes were identified in which the clinical team had commenced antibiotic therapy because of suspected VARTI. Probability of pneumonia was estimated using a modified Clinical Pulmonary Infection Score (mCPIS). Episodes meeting HELICS definitions of VAP were identified, and reasons for other episodes not meeting definitions examined. In the second period, each patient was also assessed with regards to the development of a ventilator-associated event (VAE), according to recent US definitions.

**Results:** The study included 306 invasively ventilated patients; 282 were admitted to ICU for 48 h or more. 32 (11.3%) patients were commenced on antibiotics for suspected VARTI. Ten of these episodes met HELICS definitions of VAP, an incidence of 4.2 per 1000 intubation days. In 48% VARTI episodes, concurrent chest radiography was not performed, precluding the diagnosis of VAP. Mechanical ventilation (16.0 vs. 8.0 days; *p* = 0.01) and ICU stay (25.0 vs. 11.0 days; *p* = 0.01) were significantly longer in patients treated for VARTI compared to those not treated. There was no overlap between episodes of VARTI and of VAE.

**Discussion:** HELICS VAP surveillance definitions identified less than one-third of cases in which antibiotics were commenced for suspected ventilator-associated RTI. Lack of chest radiography precluded nearly 50% cases from meeting the surveillance definition of VAP, and as a consequence we are almost certainly underestimating the incidence of VAP in Wales.

## Introduction

Pneumonia is the major cause of ICU-acquired infection in Europe and the United States (Vincent et al., 1995; Richards et al., 2000; Burgmann et al., 2010), the vast majority of episodes occurring in intubated and mechanically-ventilated patients (ventilator-associated pneumonia, VAP; Kohlenberg et al., 2010). VAP tends to occur in the sickest of critically ill patients (Chastre and Fagon, 2002; Blot et al., 2014) and is associated with appreciable morbidity, mortality, and financial cost (Safdar et al., 2005; Bekaert et al., 2011; Melsen et al., 2013; Muscedere et al., 2013). Given that some factors associated with development of VAP appear modifiable (Barbier et al., 2013), attention has been focused on regional and national healthcare-associated infection (HAI) surveillance indices to guide local quality improvement and wider bench-marking.

**Figure 1:**
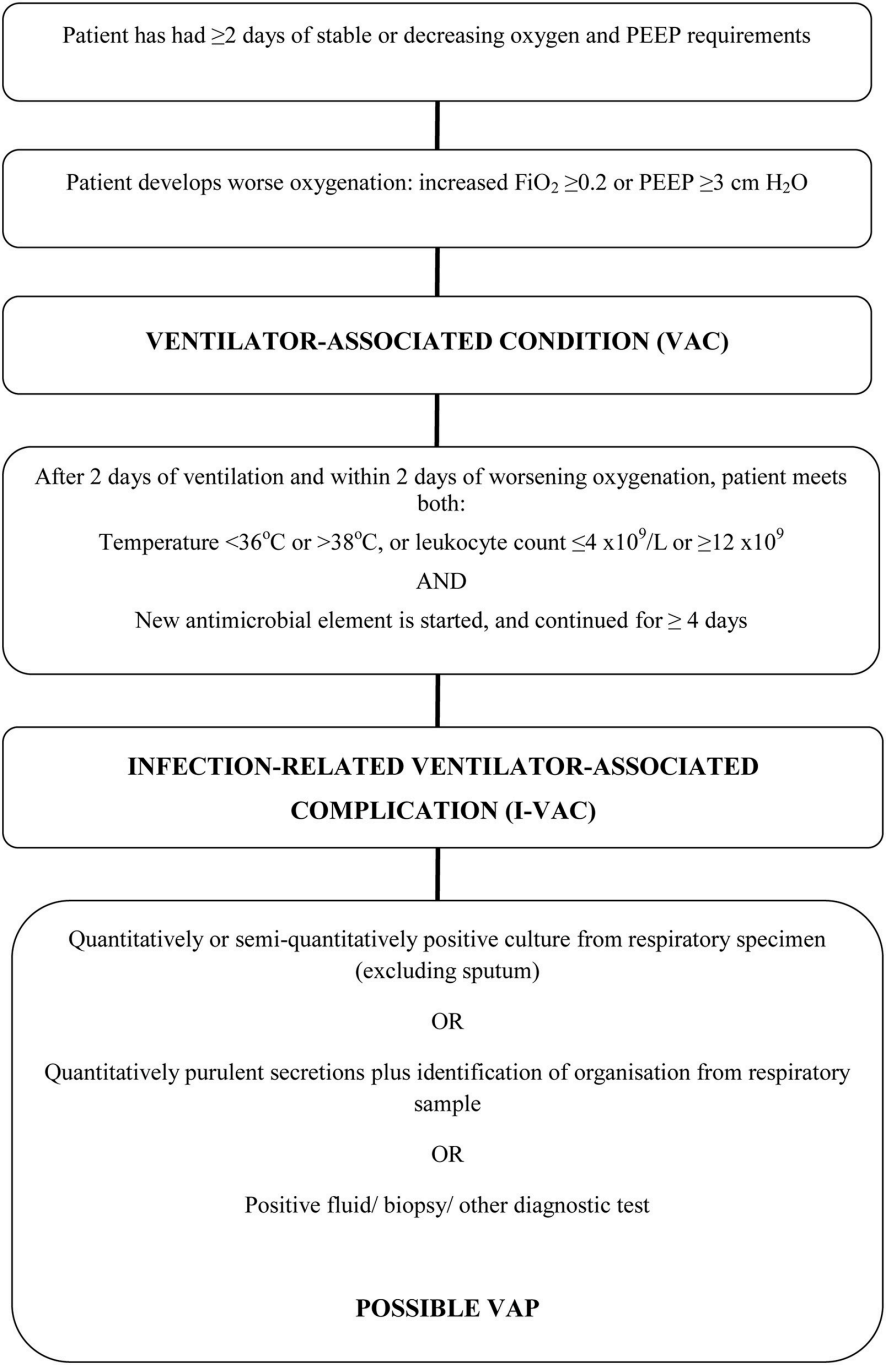
**Ventilator-associated events surveillance algorithm**. Adapted from Magill (Klompas, 2012).

The Welsh Healthcare-Associated Infection Programme (WHAIP) has monitored episodes of VAP through the National Mandatory Healthcare Associated Infection Surveillance Programme for Wales since 2008. Using European HELICS surveillance definitions (Table 1; HELICS, 2004) clinical staff in each ICU have reported VAP episodes equivalent to an incidence density of 1.2–2.2 per 1000 intubation days for 2009–2013 (WHAIP, 2014). These rates are low when compared to data from other areas of the UK (Scotland, 6.5 per 1000 intubation days; HPS/SICSAG, 2013) and Europe (12.2 per 1000 intubation days; ECDC, 2008). This may reflect the success of national healthcare improvement initiatives in Wales (e.g., the 1000 Lives Campaign), but could feasibly underestimate the true incidence of ventilator-associated pneumonia (VAP) given how subjective the clinical and radiological features of VAP can be (Klompas, 2012). In the US, loss of confidence has led to the recent revision of ICU surveillance methodology and the introduction of alternate ventilator-associated event (VAE) surveillance definitions by the Centers for Disease Control and Prevention National Healthcare Safety Network (CDC NHSN). The primary focus of these definitions is now an objective deterioration in oxygenation (Figure 1; Magill et al., 2013). Such “Ventilator-Associated Conditions” (VACs) may then potentially be sub-classified according to other unambiguous features (temperature, white cell count, and initiation of antibiotic therapy) as an Infection-related Ventilator-Associated Complication (I-VAC) for mandatory surveillance purposes.

**Table 1:** **HELICS case definition of intubation-associated pneumonia (HELICS, 2004)**.

**Criteria**	**Feature**
**Radiological**. One or, in patients with underlying cardiac or pulmonary disease, two chest x-rays or CT scans with image suggestive of pneumonia	
**Systemic**. At least one of:	1. Fever (>38°C)
	2. Leukopenia (<4 × 10^9^/L white blood cells) or leukocytosis (≥12 × 10^9^/L white blood cells)
**Pulmonary**. At least one of, or in the absence of supportive microbiological data, at least two of:	1. New onset of purulent sputum, or change in character of sputum
	2. Worsening gas exchange
	3. Cough, or dyspnoea, or tachypnoea
	4. Suggestive auscultation

*An ICU-acquired infection is one which occurs later than 48 h in ICU. Patients must fulfill radiological, systemic and pulmonary criteria to meet the HELICS definition of pneumonia. For Intubation-associated pneumonia, an invasive respiratory device must have been present for at least part of the preceding 48 h*.

Our primary aim was to identify episodes of VAP according to the HELICS definition, and to explore reasons why other episodes of ventilator-associated respiratory tract infection (VARTI) did not meet the criteria. Our secondary aims were to investigate clinical outcomes among the wider group of patients being treated for suspected VARTI, and to explore the feasibility of using alternative methods to identify infective ventilator-associated morbidity for surveillance purposes in Wales.

## Materials and methods

A prospective evaluation of current clinical practice by individual Health Boards was conducted within the mandated critical care surveillance programme in Wales. For this reason formal ethical approval was not necessary, however the Clinical Audit departments at each center approved participation.

The study was conducted in two stages. During the initial 14-day study period (November 2012), local investigators identified through the Welsh Intensive Care Society Audit and Research Group (WICSARG) were asked to complete a daily standard data collection form for each intubated and mechanically ventilated patient either already present on or admitted to their ICU. A VARTI was considered to have occurred when the clinical team commenced an invasively ventilated patient on systemic antibiotic therapy for suspected respiratory infection 48 h or more after ICU admission. For each episode, clinical and radiological features were recorded in order to make a diagnosis of pneumonia according to the HELICS definition (Table 1). The modified Clinical Pulmonary Infection Score (mCPIS) was also calculated (Table 2; Fartoukh et al., 2003) and used to give an indication of likelihood of pneumonia, with a score of 6 or more interpreted as suggesting a high probability. Microbiology results from contemporaneous respiratory specimens were also collected.

**Table 2:** **Modified clinical pulmonary score (mCPIS; Fartoukh et al., 2003)**.

**Component**	**Value**	**Points**
Temperature (°C)	≥36.5 and ≤38.4	0
	≥38.5 and ≤39.0, or ≥36.1 and ≤36.4	1
	≤36.0 or ≥39.0	2
Blood leukocytes (× 10^9^/L)	≥4 and ≤11	0
	<4 or >11	1
Tracheal secretions	Rare	0
	Abundant	1
	Abundant and purulent	2
Oxygenation	PaO_2_:FiO_2_ > 31.6 kPa	0
	PaO_2_:FiO_2_ ≤31.6 kPa	2
Chest radiograph	No infiltrate	0
	Patchy or diffuse infiltrate	1
	Localized infiltrate	2
Total		

*Modified CPIS of six or more considered to represent a high probability of pneumonia*.

The second stage of the study was undertaken between March and April 2014. During this period, ventilator settings and physiological measurements were also collected daily to enable additional identification of VAE according to NHSN CDC definitions, (see Figure 1; Magill et al., 2013). Episodes of VARTI and VAP according to the HELICS definition were identified as previously. For this stage of the study, investigators were also asked to submit 90-day outcome data (duration of mechanical ventilation and ICU length of stay, ICU, and hospital mortality) from their local WardWatcher (Critical Care Audit Ltd., UK) database.

## Statistical analysis

Our primary outcomes were: episodes of VARTI (as defined above) and of VAP, according to the HELICS definition. Secondary outcomes were: probability of pneumonia in all cases of VARTI according to a mCPIS, and episodes of VAE and 90-day outcomes (for patients in the second stage of the study). Data was collected using Microsoft Access and analyzed using MINITAB 13.32 (Minitab Inc., UK). *T*-tests were used to analyze normally-distributed data and Mann–Whitney U-test for non-parametric data. Proportions were compared using Fisher's exact test.

## Results

We collected data on 306 invasively ventilated ICU patients; of these, 282 were admitted to ICU for 48 h or more. VARTI developed in 32 (11.3%) patients (one patient developed two episodes of VARTI), a median period of nine (IQR 5–14) days after intubation and initiation of mechanical ventilation. There were no significant differences in baseline characteristics between patients developing VARTI vs. other patients (Table 3). The commonest features present when antibiotics were commenced were: abnormal white cell count and offensive and/or purulent sputum (48 and 45%, episodes, respectively), whereas deterioration in gas exchange was relatively uncommon (24% episodes; Table 4).

**Table 3:** **Baseline characteristics of patients ventilated for 48 h or more**.

	**Patients developing VARTI (*n* = 32)**	**Other patients ventilated 48 h or more (*n* = 219)**	**All**	
**PATIENTS VENTILATED FOR 48 H OR MORE—BOTH STUDY PERIODS (*****n*** = **251)**				
Age (years), mean (± *SD*)	64.7 (± 15.1)	59.7 (± 16.5)	60.4 (± 16.4)	*p* = 0.107
Sex	59.3% male	55.5% male	55.3% male	*p* = 0.703
Median intubation day at onset of VARTI (IQR)	9 (5–14)	–	–	
	**Patients developing VARTI (*n* = 15)**	**Other patients ventilated for 48 h or more (*n* = 98)**	**All**	
**PATIENTS VENTILATED FOR 48 H OR MORE—SECOND STUDY PERIOD ONLY (*****n*** = **113)**				
BMI, mean (± *SD*)	25.8 ± 3.5	26.7 ± 5.9	26.6 ± 0.5.7	*p* = 0.449
Underlying severe respiratory failure	1/15 (6.7%)	7/98 (7.1%)	8/113 (7.1%)	*p* = 0.945
Nature of surgery	Non-surgical 14/15 (93.3%)	Non-surgical 73/98 (74.4%)	Non-surgical 87/113	*p* = 0.106
	Emergency surgical 1/15	Emergency surgical 16/98	Emergency surgical 17/113	
	Elective surgical 0	Elective surgical 9/98	Elective surgical 9/113	
Primary reason for admission “Respiratory”	8/15 (53.3%)	33/98 (33.7%)	41/113 (36.3%)	*p* = 0.152
APACHE II score, mean (± *SD*)	18.3 ± 6.5	16.2 ± 7.4	16.5 ± 7.3	*p* = 0.275

**Table 4:** **Clinical and radiological features present at initiation of antibiotics for suspected VARTI**.

	**First study period (*n* = 17)**	**Second study period (*n* = 16)**	**Combined (*n* = 33)**
Abnormal white cell count	6	10	16
Abundant and/or purulent sputum	6	9	15
Abnormal temperature (<36.5 or >38.0)	1	11	12
Chest x-ray signs	8	4	12
“Deterioration in gas exchange”	3	5	8
Signs on auscultation	0	3	3
Cough	1	1	2

**Figure 2:**
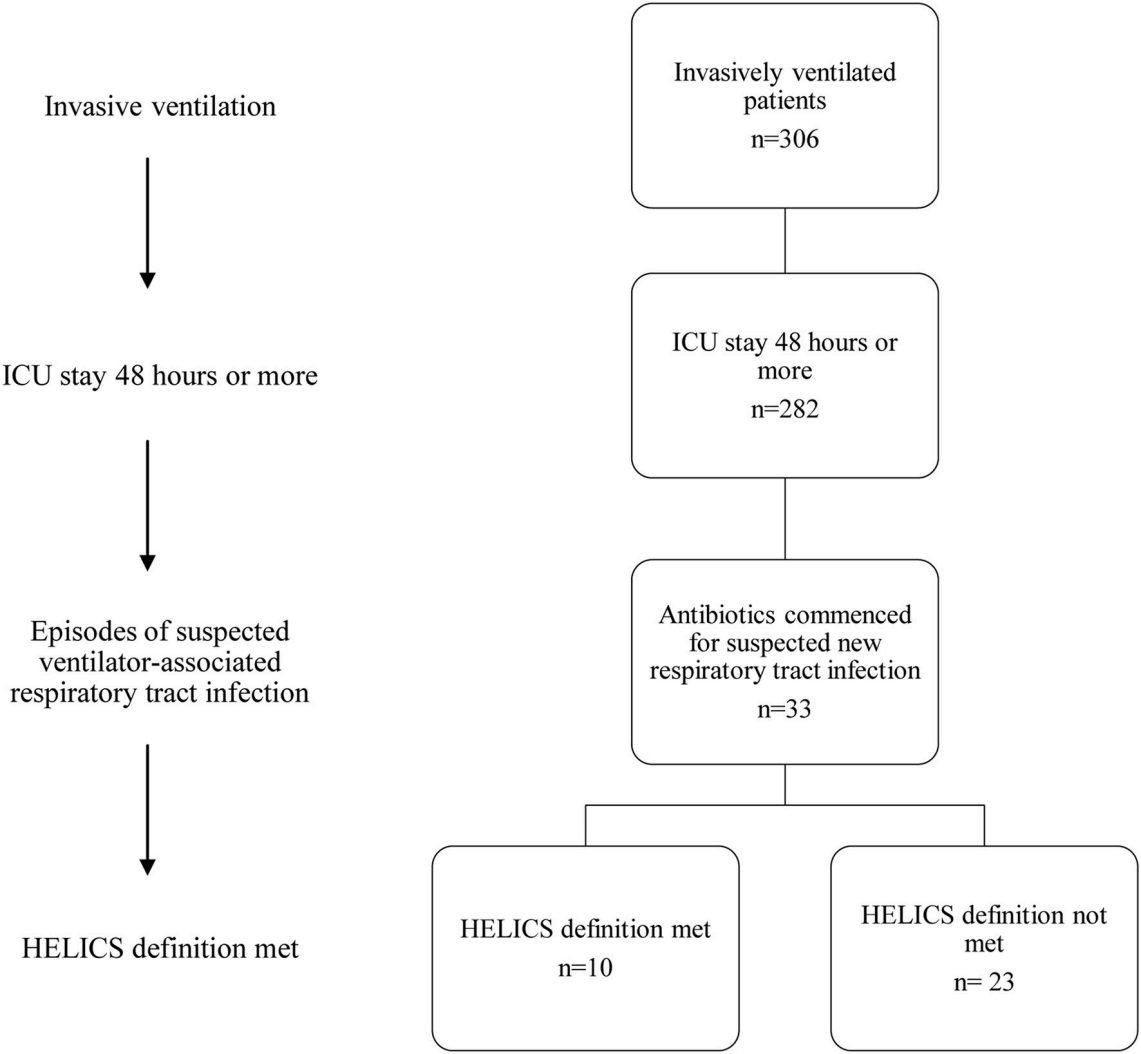
**Flowchart demonstrating numbers of patients with suspected ICU-acquired respiratory tract infection**.

Among those with VARTI, only 10 episodes met the HELICS definition for VAP (Figure 2 and Table 5). The total number of intubation days (for ventilated patients admitted to ICU for 48 h or more) was 2381, giving a VAP rate of 4.2 per 1000 intubation days. In total 3.5% of all patients admitted to ICU for 48 h or more developed a HELICS-defined VAP. Six of the 10 HELICS-defined cases of VAP were “PN1,” i.e., on the basis of a minimally-contaminated invasive respiratory sample (e.g., BAL; Table 6). The main reasons that episodes of VARTI failed to meet HELICS definitions are presented in Table 7. Most strikingly, in 16 (48.4%) cases, chest radiography was not performed on the day antibiotics were prescribed for new onset respiratory tract infection precluding diagnosis according to HELICS definition.

**Table 5:** **mCPIS and microbiological characteristics of episodes of VARTI**.

	**HELICS definition met met (*n* = 10)**	**HELICS definition not met (*n* = 23)**
Mean mCPIS (± *SD*)	6.5 (± 1.72)	3.8 (± 1.51)^*^
Positive respiratory microbiology (22/33 episodes)	– 7 out of 10 episodes: – *Enterococcus* sp. (*n* = 2) *Enterobacter* sp. (*n* = 1) – *Staphylococcus aureus* (flucloxacillin sensitive) (*n* = 1) – *Klebsiella* sp. (*n* = 1) – *Candida* sp. (*n* = 2) – “Other Gram-negative organism” (*n* = 1)	– 15 out of 23 episodes: – *Enterobacter* sp. (*n* = 1) – *Enterococcus* sp. (*n* = 1) – *Escherichia coli* (*n* = 1) – *Klebsiella* sp. (*n* = 4) – *Staphylococcus aureus* – (flucloxacillin sensitive) (*n* = 2) – *Pseudomonas* sp. (*n* = 1) – *Stenotrophomonas maltophilia* (*n* = 1) – *Candida* sp. (*n* = 3) – mixed growth (*n* = 1)

* *p = 0.01*.

**Table 6:** PN classification of HELICS-defined episodes of VAP (HELICS, 2004) and “non-HELICS” episodes referred to as “PN 0”^*^.

**Classification of episode**	**Number of episodes**	**Organism**	**Mean mCPIS**
“PN0”	6	*Candida* sp.	4.2
		*Enterobacter* sp.	
		*Klebsiella* sp.	
		“Other Gram-negative organism”	
		Mixed growth	
PN1	6	*Staphylococcus* sp.	6.7
		*Candida* sp.	
		“Other Gram-negative organism”	
		Mixed growth	
PN2	1	*Enterococcus* sp.	4.0
PN3	0		
PN4	0		
PN5	3	By definition none identified	7.0

* “PN 0,” Chest radiography not performed at time of initiation of antibiotic therapy for suspected respiratory-tract infection onset 48 h or more after onset ventilation, together with [two of: diagnostic signs (temperature, abnormal white cell count) OR symptoms (sputum, cough dyspnoea, chest signs, worse gas exchange)] AND (positive microbiology);

PN 1, Bacteriologic diagnosis on basis of quantitative analysis of minimally contaminated lower respiratory tract specimen (e.g., broncho-alveolar lavage);

PN 2, Bacteriologic diagnosis on basis of quantitative culture from possibly contaminated lower respiratory tract specimen (e.g., endotracheal aspirate);

PN 3, Alternative microbiological diagnosis (e.g., blood culture, culture of pleural fluid);

PN 4, Positive sputum culture or non-quantitative lower respiratory tract specimen culture;

*PN 5, No positive microbiology*.

**Table 7:** **Reasons that episodes of VARTI did not meet HELICS definition (HELICS definition not met in 23 out of 33 VARTI episodes)**.

**Criteria**	**Not met**
Chest x-ray not performed	16
Chest x-ray performed but did not indicate new infiltrates	5
Lack of systemic signs	8
Lack of pulmonary signs	6
Lack of microbiological evidence	6

Of the 10 HELICS-defined cases of VAP, seven had mCPIS of six or more (suggesting high probability of pneumonia). In the 23 episodes in which HELICS criteria were not fulfilled only two had a mCPIS score of six or more; mean mCPIS was significantly lower among this cohort of patients [3.8 vs. 6.5 (*p* = 0.01); Table 5], though positive microbiology was subsequently identified in approximately two-thirds of cases. In six of these 23 non-HELICS episodes, there were two or more signs and symptoms suggestive of pneumonia as well as subsequent positive microbiology in the absence of chest x-ray examination, we have referred to these episodes as “PN0” (Table 6).

**Figure 3:**
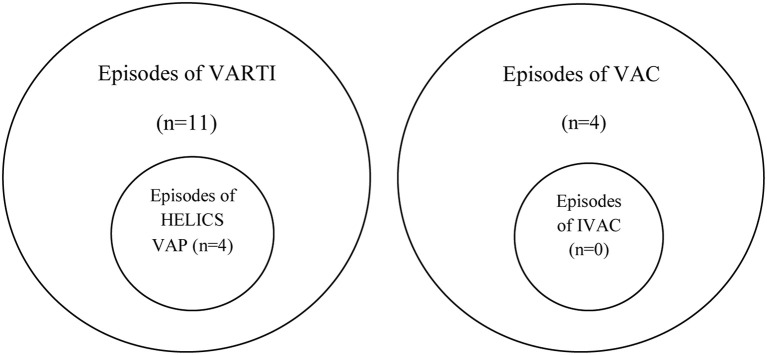
**Euler diagram illustrating episodes of VARTI, HELICS VAP, and VAC**.

Applying new CDC NHSN criteria, we identified four cases of ventilator-associated complication (VAC) among the 113 patients ventilated for 48 h or more in the second cohort: three on the basis of as a result of sustained increase in PEEP application and one because of sustained increase in inspired oxygen concentration (Table 8). In none of these cases could an episode of VAC be further sub-classified as an IVAC. There was no overlap between cases of VARTI and VAC (see Euler diagram, Figure 3).

**Table 8:** **Baseline characteristics of patients who did and did not develop VAC**.

	**Patients ventilated for minimum 48 h who develop VAC (*n* = 4)**	**Patients ventilated for minimum 48 h who not develop VAC (*n* = 109)**
Age, mean (± *SD*)	57.5 ± 15.6	59.1 ± 17.5
Sex	50% male	57.1% male
BMI, mean (± *SD*)	35.3	26.2^*^
Presence of underlying severe respiratory disease	1 (25%)	7 (6.4%)
Nature of surgery	3 (75%) Non-surgical	84 (77%) Non-surgical
	1 Emergency/urgent surgical	16 Emergency/urgent surgical
		8 Elective/scheduled surgical
Median intubation day at VAC onset (IQR)	6 (3.8–13.5)	–
Respiratory cause of primary reason for admission	1/4 (25%)	40/109 (36.7%)
APACHE II, mean (± *SD*)	22.5 ± 17.1	16.3 ± 6.7

* *Data missing in four cases*.

With regards to clinical outcomes (measured only in this second stage), initiation of antibiotic therapy for VARTI 48 h or more after onset of mechanical ventilation was associated with significantly prolonged mechanical ventilation and ICU stay [median 16.0 vs. 8.0 days (*p* = 0.01) and 25.0 vs. 11.0 days (*p* = 0.01), respectively], though not a significant increase in ICU or hospital mortality (Tables 9, 10). Sample sizes were too small to compare outcome data between patients who did and did not develop VAP or VAC.

**Table 9:** **Outcomes: durations of mechanical ventilation and ICU length of stay, according to ICU-acquired respiratory morbidity**.

**Respiratory morbidity**	**Duration of mechanical ventilation among sub-groups (days)**			
	**Present**	**Absent**	**All**
VARTI Median (IQR)	16.0 (9–49)	8.0 (3.0–18.3)	*p* = 0.01	9.0 (3.0–19.0)
HELICS-defined VAP Median (IQR)	13.5 (9.75–17.25)	9.0 (3.0–20.0)		
“PN 0” VAP Median	40.5	–		
VAC Median (IQR)	15.5 (8.25–28.75)	9.0 (3.0–19.5)		
**Respiratory morbidity**	**Duration of ICU stay among patient sub-groups (days)**			
	**Present**	**Absent**		**All**
VARTI Median (IQR)	25.0 (11.0–49.0)	11.0 (4.0–26.5)	*p* = 0.01	12.4 (5.6–27.6)
HELICS-defined VAP Median (IQR)	18.5 (10.0–25.5)	11.5 (5.0–28.0)		
“PN 0” VAP Median	48.5	–		
VAC Median (IQR)	15.5 (7.75–36.0)	12.5 (5.0–28.0)		

**Table 10:** **Outcomes: survival to ICU and hospital discharge, according to ICU-acquired respiratory morbidity**.

**Respiratory morbidity**	**ICU survival among patient sub-groups**			
	**Present**	**Absent**		**All**
VARTI	12/15 (80%)	70/98 (68.4%)	*p* = 0.45	82/113 (72.6%)
HELICS-defined VAP	3/4 (75%)	79/109 (72.5%)		
“PN 0” VAP	1/2 (50%)	–		
VAC	1/4 (25%)	81/109 (74.3%)		
**Respiratory morbidity**	**Hospital survival among patient sub-groups**			
	**Present**	**Absent**		**All**
VARTI	12/15 (80%)	67/ 98 (68.4%)	*p* = 0.265	79/113 (69.9%)
HELICS-defined VAP	3/4 (75%)	75/109 (68.8%)		
“PN 0” VAP	1/2 (50%)	–		
VAC	1/4 (25%)	76/109 (69.7%)		

## Discussion

This study sheds significant light on the diagnostic processes undertaken by ICU clinicians in Wales prior to commencing antibiotics for suspected VARTI and provides new insight into the utility of the currently adopted HELICS criteria used for surveillance.

Our study investigated 282 invasively ventilated patients admitted to ICU for 48 h or more, and in this group antibiotics were commenced for suspected VARTI in 32 (11.3%) patients. The most common clinical features present when clinicians commenced antibiotic therapy were abnormal white cell count and abundant and/or purulent sputum, rather than deterioration in gas exchange which has been the focus of new CDC NHSN surveillance methods. Initiation of antibiotic therapy for suspected VARTI was associated with an approximate doubling of duration of mechanical ventilation and ICU stay.

There were 10 episodes of VAP according to HELICS definitions, equating to 4.2 episodes per 1000 intubation days. Appreciating the relatively small scale of the study, that a “Hawthorne effect” may have influenced the reporting practices of clinical staff, and that inter-rater variation in the interpretation of data will inevitably lead to variation in reported incidence (Thomas et al., 2011; Stevens et al., 2014), the VAP incidence reported during this period of enhanced surveillance is closer to the incidence reported by colleagues in Scottish and European centers than previous Welsh surveillance program estimates. It seems possible that episodes of VAP have been historically under-reported in Wales.

The most striking finding of our study is the lack of consistent approach to chest radiography, though. Clinicians did not perform chest radiography in nearly half of the cases treated for suspected VARTI, and this is important since chest radiography is used to define VAP for purposes of clinical therapy as well as for surveillance. Antibiotic stewardship principles indicate that the minimum effective duration of antibiotic therapy should be used (Kollef and Micek, 2012). If ventilator-associated tracheobronchitis (VAT) is to be treated with antibiotic therapy—and recent evidence seems to suggest that this may prevent progression to VAP and improve clinical outcomes (Nseir et al., 2005, 2008, 2014; Shahin et al., 2013; Martin-Loeches et al., 2015)—clinical resolution may occur earlier with treatment of VAT than with VAP (Martin-Loeches et al., 2015). The distinction between episodes of VARTI with and without radiographic evidence of consolidation (i.e., between episodes of VAP and VAT) is therefore likely to be important clinically.

However, for surveillance purposes (according to HELIS), chest radiography features are essential for defining an episode of VAP. Given the variability in chest x-ray performance, it is perhaps unsurprising that HELICS-defined episodes of VAP represented less than one-third of cases of the total actually treated for VARTIs in our study. Subjectivity and inter-observer variability with respect to chest radiography interpretation have been highlighted as a potential contribution to differences in reported VAP rates in the US (Klompas, 2012) but we are unaware of previous reports which describe the non-performance of chest radiography as being such a potentially important confounder of surveillance data.

In the US, attempts to focus on “hard” objective data and to exclude more subjective elements (including chest radiography; Klompas, 2010) recently led to complete revision of ICU surveillance methodology and the introduction of the concept of a “Ventilator-Associated Event,” defined on the basis of a deterioration in oxygenation as a “Ventilator-Associated Complication” or “VAC” (Magill et al., 2013). An episode of VAC correlates with poorer clinical outcome (Klompas et al., 2011), but a number of authors have observed that such episodes represent a heterogeneous group of pulmonary conditions which include VAP but also atelectasis, pulmonary edema, and thrombo-embolism (Lilly et al., 2014; Stoeppel et al., 2014; Bouadma et al., 2015; Chang et al., 2015). In our cohort, only a very small number of cases of VAC were identified. Mortality was high in the group of patients with VAC but we found no overlap with our VARTI cohort. Deterioration in oxygenation does not appear to be a frequent reason for commencing antibiotic therapy in our patients. Furthermore, in ICUs which predominantly use paper-based rather than electronic patient records, daily collection of data for VAC analysis was reported by our data collectors to be rather difficult and time-consuming.

We calculated a modified CPIS when antibiotics were commenced for suspected respiratory tract infection as an index of the probability of pneumonia. A mCPIS of six or greater was significantly more likely to occur with HELICS-defined episodes of VAP and for other episodes of RTI. However, as chest x-ray features contribute up to two points to this score, and are also an essential criterion for HELICS-confirmed episode, this is an expected association. Without reducing the variability with which chest radiography is performed, CPIS is unlikely to provide a reliable tool for our surveillance program.

Initiation of antibiotic therapy for suspected VARTI was associated with a significant increase in duration of mechanical ventilation and ICU stay. Among this group of patients were those with HELICS-defined episodes of VAP, for whom longer durations of ventilation and ICU stay are consistent with previous work (Safdar et al., 2005; Bekaert et al., 2011; Melsen et al., 2013; Muscedere et al., 2013). However, among this group were also cases in which there was positive respiratory microbiology and two or more diagnostic signs or symptoms of pneumonia, but in whom chest radiography had not been performed. Not having met established HELICS definitions, we have referred to these as “PN0” episodes (Tables 6, 9, 10). Our present limited data suggests that these patients also have relatively prolonged duration of mechanical ventilation and ICU stay and suggests to us that the application of HELICS surveillance definition is failing to detect episodes of VARTI with appreciable morbidity.

Our study has a number of limitations. Firstly, data collection was restricted to two 14-day periods. Infectious episodes occurring outside the primary data collection period may have been missed, and as a result we may have under-estimated the number of infections occurring on a per patient basis, rather than per intubation days. Despite this, our estimate of VARTI (11.3%) correlates very closely with estimates from a previous UK antimicrobial point prevalence study (12%; Coello et al., 2011). Secondly, with the exception of the VAE data during the latter phase of the study, data collection was triggered by initiation of antibiotic therapy. Significant respiratory tract infection could have been present, but our ability to capture an episode relied on the clinical team initiating treatment. However, using the initiation of antibiotics to trigger ICU surveillance data capture has previously been described (Kaiser et al., 2014) and is recognized in the VAE surveillance methodology as an unambiguous factor within the definition of an “I-VAC.” A major strength of our study is that all acute ICUs in Wales contributed and we believe our findings accurately reflect current intensive care practice.

## Conclusions

Our data suggest that established measurements of VAP rates in Wales have unfortunately under-estimated the true incidence of VAP. The higher rate suggested by our enhanced surveillance study is more in keeping with rates published for comparable geographical regions. However, even this higher rate may underestimate the true incidence given the marked variability in performance of chest x-ray at time of diagnosis of respiratory tract infection. The absence of radiographic data is a limitation to a surveillance mechanism which uses the HELICS definition of VAP. This is a novel finding and a concern which may be relevant outside our country.

Ensuring a mechanism for identifying the wider burden of VARTI in Wales while enabling us to contribute to a Europe-wide surveillance programme will be a challenge, particularly in the context of current diagnostic practices. The use of mCPIS for surveillance is hampered by inconsistent radiographic performance and our study indicates that mCPIS could not reliably replace the HELICS definition of VAP. Our results also confirm that adopting the CSC NHSN VAE surveillance methodology in Wales may not provide a solution. The low degree of agreement between episodes of clinician-perceived respiratory tract infection and episodes of VAC among our patient population—as well as logistical problems of data collection in “paper based” ICUs—raises doubt over the suitability of this methodology in Wales at present. However, a sensible step may be the further characterization of episodes of VARTI—diagnosed according to clinical and microbiological features, but without the benefit of radiographic data. This approach including the “PN0” definition is being piloted in the Welsh ICUs and the validity of this approach should be subject to further collaborative study.

## Author contributions

RP, WH, SH, and TS co-designed the study, coordinated data collection, performed data analysis, and contributed to this draft. HR and GS co-designed the study, coordinated data collection, and contributed to this draft. All authors have agreed to be considered accountable for this work and approve the submitted draft.

### Conflict of interest statement

The authors declare that the research was conducted in the absence of any commercial or financial relationships that could be construed as a potential conflict of interest.
